# Usefulness of apolipoprotein B-depleted serum in cholesterol efflux capacity assays using immobilized liposome-bound gel beads

**DOI:** 10.1042/BSR20190213

**Published:** 2019-04-02

**Authors:** Yuna Horiuchi, Ryunosuke Ohkawa, Shao-Jui Lai, Shitsuko Shimano, Michio Hagihara, Shuji Tohda, Takahiro Kameda, Minoru Tozuka

**Affiliations:** 1 Analytical Laboratory Chemistry, Graduate School of Medical and Dental Sciences, Tokyo Medical and Dental University (TMDU), 1-5-45 Yushima, Bunkyo-ku, Tokyo 113-8519, Japan; 2 Analytical Laboratory Chemistry, Graduate School of Health Care Science, Tokyo Medical and Dental University, 1-5-45 Yushima, Bunkyo-ku, Tokyo 113-8519, Japan; 3 Clinical Laboratory, Medical Hospital, Tokyo Medical and Dental University (TMDU), 1-5-45 Yushima, Bunkyo-ku, Tokyo 113-8519, Japan; 4 Department of Medical Technology, School of Health Sciences, Tokyo University of Technology, 5-23-22 Nishikamata, Ota-ku, Tokyo 144-8535, Japan

**Keywords:** apolipoprotein B-depleted serum, cardiovascular disease, cholesterol efflux capacity, high-density lipoprotein

## Abstract

Cholesterol efflux capacity (CEC) in atherosclerotic lesions is the main anti-atherosclerotic function of high-density lipoprotein (HDL). In recent studies, apolipoprotein (apo) B-depleted serum (BDS) obtained with the polyethylene glycol (PEG) precipitation method is used as a cholesterol acceptor (CA) substitution for HDL isolated by ultracentrifugation. However, the suitability of BDS as a CA is controversial. In the present study, CEC obtained from BDS (BDS-CEC) was evaluated based on a parameter, defined as whole-CEC, which was calculated by multiplying CEC obtained using fixed amounts of HDL by cholesterol concentration to HDL-cholesterol (HDL-C) levels in the serum. Significant correlation (r = 0.633) was observed between both CECs. To eliminate systematic errors from possible contamination with serum proteins and low-density lipoprotein (LDL) or very-LDL (VLDL) in BDS-CEC, the deviation of each CEC-BDS from the regression equation was compared with serum protein, LDL, and triglyceride (TG) levels. No correlation was observed between the deviation and the levels of each of these serum components, indicating that the deviations do not derive from systematic error. Further, to evaluate the effects of serum protein on the results, we measured BDS-CEC of reconstituted serum samples prepared using combinations of five levels of serum proteins with five levels of HDL-C. No significant change in BDS-CEC was observed in any combination. These results indicate that BDS-CEC reflects not only the function of HDL but also its concentration in serum.

## Introduction

Cardiovascular disease (CVD) is one of the leading causes of death in developed countries [1]. In most cases, there are no symptoms until a crucial event occurs, indicating that the risk prediction for CVD events using regular blood tests is important. One of the blood biomarkers is high-density lipoprotein (HDL) because of its capacity to protect against atherosclerosis, the cause of CVD [2]. In fact, serum HDL-cholesterol (HDL-C) levels have been shown to be a biomarker of CVD risk over a long period [3]. However, a recent epidemiological study showed that HDL-C does not always predict CVD risk because it indicates just the amount of HDL [4]. Therefore, the need for evaluating the function as well as the amount of HDL has been emphasized.

Cholesterol efflux capacity (CEC) is the main anti-atherosclerotic function of HDL. The atherosclerotic lesion is formed by the accumulation of foam cells, which originate from macrophages that engorged a large amount of cholesterol. HDL removes the enriched cholesterol from the foam cells and contributes to reduce the atherosclerotic lesion. Therefore, the evaluation of CEC has been proposed as an additional CVD risk marker [5].

In the first step to estimate the CEC of HDL, isolation of serum HDL is required, and the ultracentrifugation method has been prevalently used for a long time [6]. However, this method is inadequate for HDL separation in the clinical laboratory because it takes more than 2 days and demands expensive special equipment. In addition, the HDL fraction is generally concentrated but not at the same rate during ultracentrifugation and does not reflect its concentration in the serum [6,7]. Each HDL sample should therefore be adjusted to the original protein or cholesterol levels before use in the CEC assay. Another method to isolate the HDL fraction that reflects its serum concentration can be used in the CEC assay instead of ultracentrifugation. One of the candidates for this purpose is the precipitation method, in which apolipoprotein (apo) B (apoB)-containing lipoproteins are precipitated with polyethylene glycol (PEG) or a polyanion–divalent cation combination to obtain apoB-depleted serum (BDS). BDS includes the whole-HDL in a certain volume, making the amount correction of the ultracentrifugation method unnecessary. This simple method has already been used for CEC assays in several studies [8–11]. Although this method can be performed within 1 h and does not need special equipment, there are still several issues to be solved to use CEC assays for clinical applications. In addition, data obtained from CEC assays using the HDL fraction and BDS could require a different interpretation. However, a detailed comparison between these assays has not been performed. BDS contains most serum proteins, such as albumin, and contamination of the apoB-containing lipoprotein fraction with other proteins cannot be avoided because PEG and polyanion–divalent cation combination methods are not specific for apoB-containing lipoprotein [12]. Although many clinical studies have already been conducted using BDS, a thorough examination of these effects has not been performed [5,13,14]. Therefore, confirmation of whether BDS can be used as a substitute for HDL in CEC assays is urgently required. The present study aims to evaluate the availability of BDS for CEC assays. Using our novel, recently developed immobilized liposome-bound gel beads (ILG) method [15], we demonstrate that BDS obtained with PEG enables CEC assays to be easily and accurately introduced into laboratory medicine.

## Materials and methods

### Chemicals

Unless otherwise stated, all reagents were purchased from FUJIFILM Wako Pure Chemical Corporation (Osaka, Japan).

### Serum samples

Anonymized residual serum samples of patients with varying HDL-C levels were randomly obtained from the Clinical Laboratory of Medical Hospital at the Tokyo Medical and Dental University. Serum samples with icterus, aspartate aminotransferase activity > 30 U/l, and alanine aminotransferase activity > 42 U/l in males and >23 U/l in females were excluded. Serum samples of healthy subjects were obtained from volunteers who had given written informed consent at the Tokyo Medical and Dental University and used as pooled sera. These samples were stored at −80°C until use. The present study was approved by the Institutional Research Ethics Committee of the Faculty of Medicine, Tokyo Medical and Dental University [M2015-546 and M2016-049]. The present study has been performed in accordance with the World Medical Association Declaration of Helsinki.

### Isolation of HDL and lipoprotein-free fractions

HDL (1.063 < d < 1.210 g/ml) and lipoprotein-free (lipo-free) (d > 1.210 g/ml) fractions were isolated from pooled sera of healthy volunteers using ultracentrifugation as previously described [6]. HDL was similarly obtained from patient sera.

### Preparation of BDS

BDS was prepared as previously described [16]. Briefly, 16 μl of 20% PEG (molecular weight 6000) in 200 mM glycine buffer (pH 7.4) was added into 40 μl of serum followed by mixing and incubating at 22°C for 20 min. The mixture was then centrifuged at 12000×***g*** for 30 min and the supernatant was used as BDS.

### Measurement of serum lipids and proteins

Total cholesterol (TC), HDL-C, low-density lipoprotein (LDL)-cholesterol (LDL-C), and triglyceride (TG) levels were determined using the corresponding commercial kits (Kyowa Medex Co., Tokyo, Japan). Total protein (TP) levels in serum was measured using L-Type Wako TP (FUJIFILM Wako Pure Chemical Corporation, Osaka, Japan). TP levels in HDL and lipo-free fractions were determined using Lowry’s method. TP levels of HDL in the serum were calculated by multiplying the TP/TC ratios in the isolated HDL to the HDL-C levels.

### Preparation of ILGs

ILGs were prepared as previously described [15], with minor modifications. Briefly, egg lecithin (10.6 mg) and cholesterol (2.3 mg) were dissolved in 12 ml chloroform, and 30 μl of 0.5 mM 4,4-difluoro-4-bora-3a,4a-s-indacene-labeled cholesterol (BODIPY-cholesterol; Avanti Polar Lipids, Alabaster, AL) in chloroform was added to the solution. The lipid film formed under N_2_ gas was dissolved in ether and the solvent removed by evaporation. After performing this step twice, the lipid film was completely dried under N_2_ gas and suspended in 14 ml of 10 mM Tris/HCl (pH 7.4) containing 150 mM NaCl and 1 mM EDTA-2Na (Buffer A). Dried Sephacryl S-300 gel beads (0.7 g; GE-Healthcare, Tokyo, Japan) were then added to the liposome suspension, followed by swelling for 30 min at room temperature (RT). The mixture was subjected to seven cycles of freezing (−80°C) and thawing (in water at RT) to induce the formation of large multilamellar vesicles in the Sephacryl S-300 beads. Finally, the gel was washed with Buffer A, centrifuged, and resuspended in 10 ml of Buffer A. The gel suspension was stored in the dark at 4°C.

### Cholesterol efflux assay

Cholesterol efflux assay was performed using the ILG’s method as previously described [15]. Briefly, after uniformly suspending the stored ILGs, 100 μl of the ILG was aliquoted into a 2-ml Eppendorf tube. Then, 150 μl of various types of cholesterol acceptor (CA) solution as described below or Buffer A was added to the ILG followed by incubation in the dark at RT for 16 h. The mixture was resuspended by vortexing and then centrifuged. The fluorescence intensity of the supernatant was measured (Ex: 485 nm, Em: 538 nm). The values were normalized using a reference BDS evaluated in every measurement, and the ratio was represented as CEC. All samples were assayed in triplicate. In the experiments using patient serum, the CA solution was adjusted to a final concentration of 20 μg TP/ml or 10 μg TC/ml of HDL, or the BDS equivalent to 2% in the serum. The CEC measured in each condition was hereafter described as HDL-CEC (TP), HDL-CEC (TC), and CEC obtained from BDS (BDS-CEC), respectively. HDL-CEC (TP) and HDL-CEC (TC) were further converted into whole-CEC (TP) and whole-CEC (TC), respectively, using the following formulas:

Whole - CEC (TP) = HDL - CEC (TP) × serum HDL protein levels(μg/ml)/20

Whole - CEC (TC) = HDL - CEC (TC) × serum HDL cholesterol levels (μg/ml)/10

### Statistics

Correlation analyses were performed using Pearson’s test. Results of CEC values in reconstituted serum were expressed as mean ± S.D. and the tendencies were evaluated using Jonckheere–Terpstra test. Differences were considered significant at *P*<0.05.

## Results

### Correlations of HDL-CEC (TP) and HDL-CEC (TC) with HDL-C

**Figure 1:**
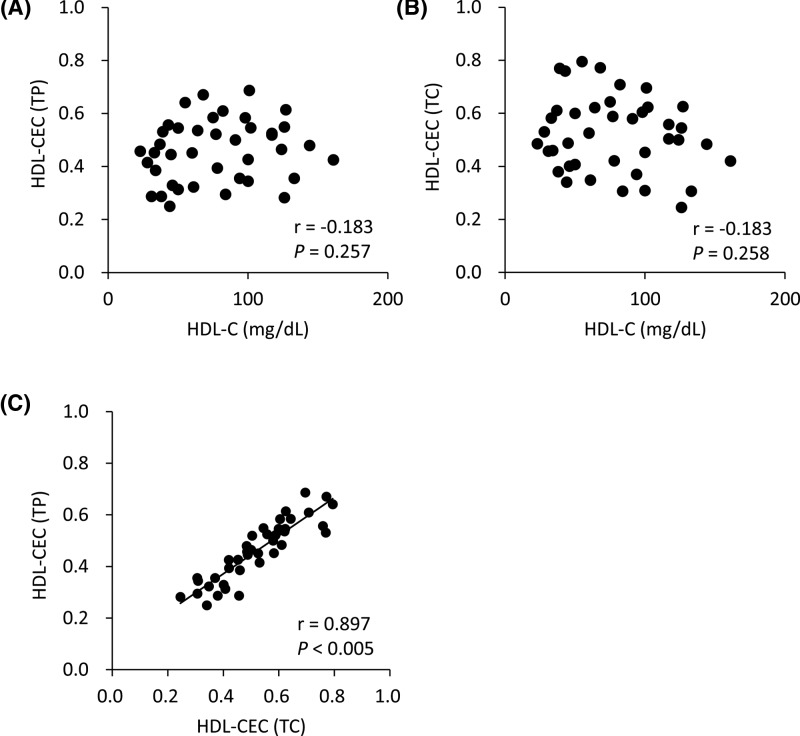
Correlations of HDL-CEC (TP) and HDL-CEC (TC) with HDL-C CECs of 40 patients’ HDL were compared with HDL-C. CECs were measured using HDL as CA after adjusting their concentrations to 20 mg TP/ml (HDL-CEC (TP)) (**A**) and 1 mg TC/dl (HDL-CEC(TC)) (**B**). HDL-CEC (TP) and HDL-CEC (TC) were also compared (**C**). Correlations were evaluated using Pearson’s test. All samples were assayed in triplicate.

CECs obtained from fixed concentrations of HDL isolated from 40 patients with various HDL-C levels (Table 1) were compared with HDL-C levels, a traditional serum biomarker for CVD development. As shown in Figure 1A,B, neither HDL-CEC (TP) (r = −0.183, *P*=0.257) nor HDL-CEC (TC) (r = −0.183, *P*=0.258) correlated with HDL-C, indicating that CEC in each HDL is unrelated with total HDL-C level. On the other hand, a significant correlation was observed between HDL-CEC (TP) and HDL-CEC (TC) (r = 0.897, *P*<0.005) (Figure 1C), suggesting that the ratio of TP to TC in most of HDL was similar.

**Table 1:** Serum lipid and protein profiles of 40 patients

	HDL-C (mg/dl)	LDL-C (mg/dl)	TG (mg/dl)	TP (g/dl)
Mean	78	106	87	7.0
S.D.	37	29	50	0.6
Min	23	56	33	5.0
Max	161	198	268	8.6
				*n*=40

### Correlation of whole-CEC (TP) and whole-CEC (TC) with HDL-C

**Figure 2:**
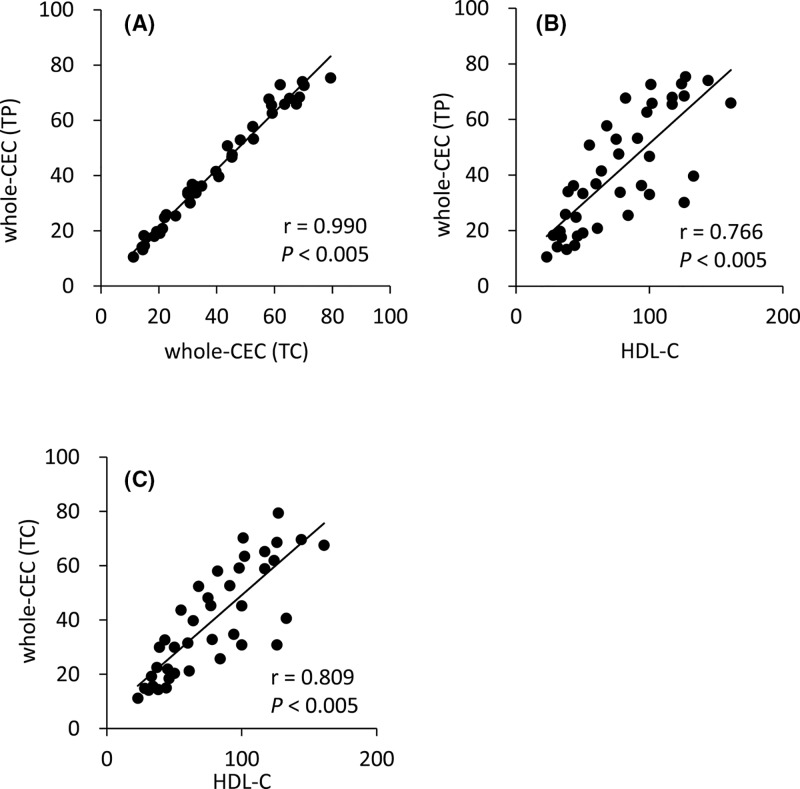
Correlation of whole-CEC (TP) and whole-CEC (TC) with HDL-C CEC of whole HDL fractions was calculated in two ways and compared (**A**). BDS-CEC was measured and compared with whole CEC (TP) (**B**) and whole CEC (TC) (**C**). Correlations were evaluated using Pearson’s test. *n*=40. All samples were assayed in triplicate.

CECs from quantitatively defined whole-CEC (TP) and whole-CEC (TC) were calculated as described in ‘Materials and methods’ section. Understandably, whole-CEC (TP) showed a strong correlation with whole-CEC (TC) (Figure 2A, r = 0.990, *P*<0.005). Both whole-CECs were also compared with HDL-C levels. Significant correlations were observed between HDL-C and whole-CEC (TP) (Figure 2B, r = 0.766, *P*<0.005) and whole-CEC (TC) (Figure 2C, r = 0.809, *P*<0.005), whereas large variations from the regression equation were observed in subjects with higher levels of HDL-C. This result indicates that whole CEC calculated by multiplying CEC per unit amount of HDL and quantity of HDL shows similar trend to HDL-C but not always consistent.

### Correlations between BDS-CEC and whole-CEC

**Figure 3:**
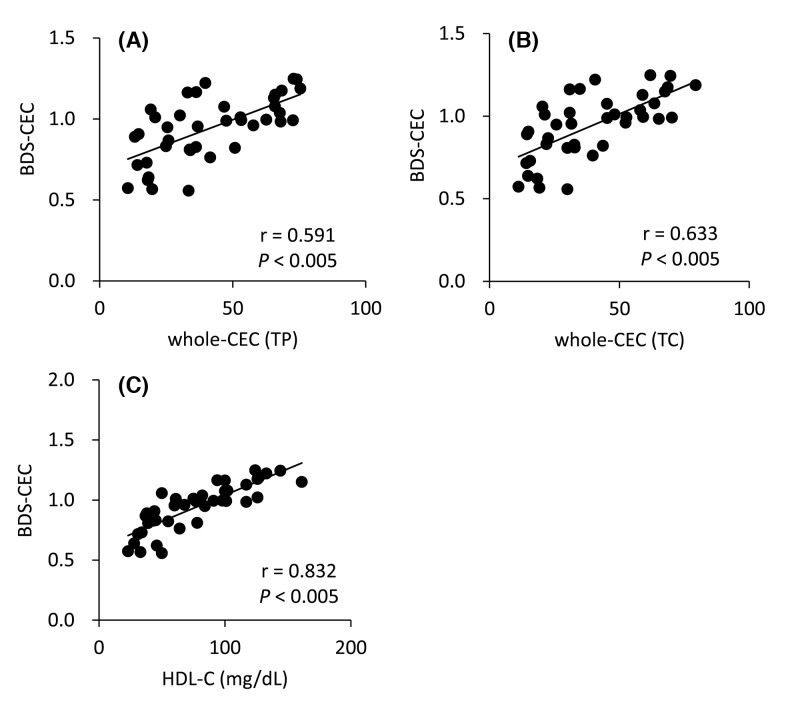
Correlation between BDS-CEC and whole CEC BDS-CEC was compared with whole CEC (TP) (**A**), whole CEC (TC) (**B**) and HDL-C (**C**). Whole CEC (TP) was calculated by serum HDL protein levels and CECs were measured using HDL as CA after adjusting their concentrations to 20 mg TP/ml, and whole CEC (TC) was calculated by serum HDL cholesterol levels and CECs were measured using HDL as CA after adjusting their concentrations to 1 mg /TC/dl as fomulas described in method. Correlations were evaluated using Pearson’s test. *n*=40. All samples were assayed in triplicate.

Two types of whole-CECs were compared with BDS-CECs. BDS-CEC showed significant correlation with both whole-CEC (TP) (r = 0.591, *P*<0.005) and whole-CEC (TC) (r = 0.633, *P*<0.005) (Figure 3A,B). This result suggests that CEC determined by using BDS reflects whole efflux capacity of HDL in serum. On the other hand, a stronger correlation was observed between BDS-CEC and HDL-C (Figure 3C, r = 0.832, *P*<0.005), whereas some samples with similar concentrations of HDL-C corresponded to clearly different BDS-CEC. The maximum variation in CEC between two samples with the same HDL-C concentration (50 mg/dl) was 0.5 (BDS-CECs of the two samples were 0.558 and 1.058).

### Effects of serum TP levels on BDS-CEC

**Figure 4:**
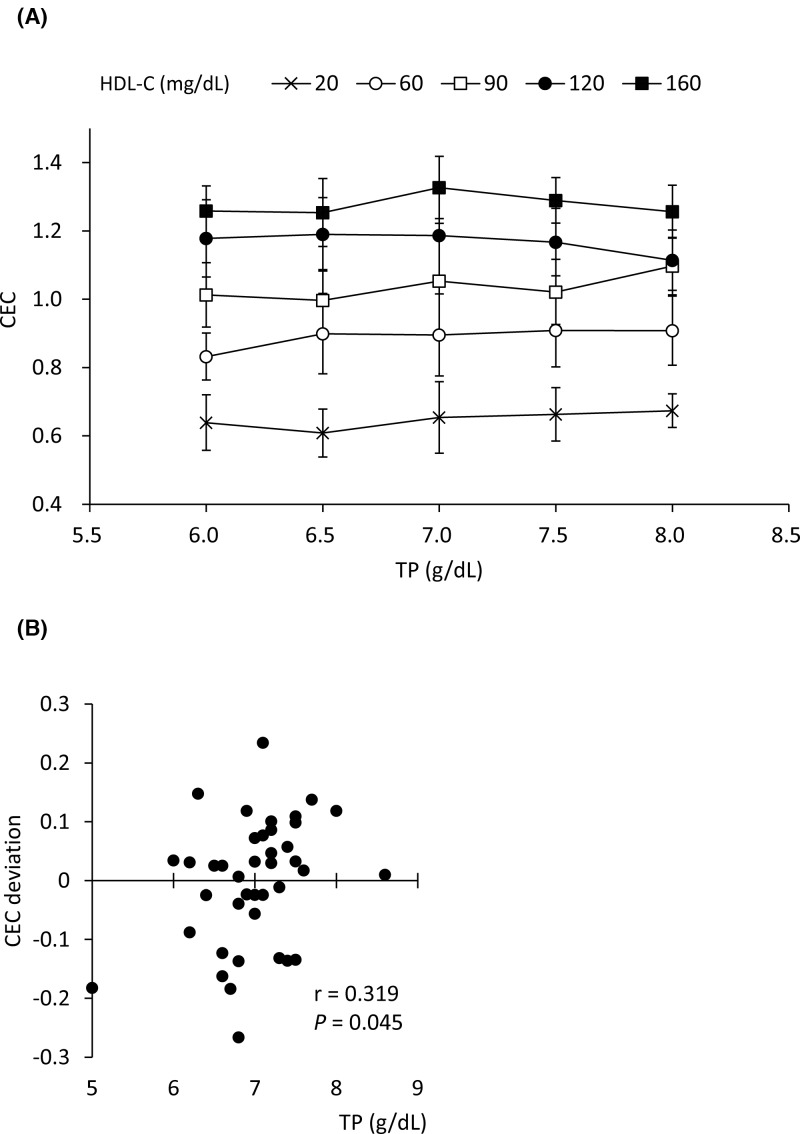
Effects of serum TP levels on BDS-CEC CEC were measured using BDS obtained from reconstituted serum with indicated TP and HDL-C levels as CA (**A**). Results are expressed as mean ± S.D. No significant trend was observed in any HDL-C concentration using the Jonckheere–Terpstra test. (**B**) Deviation of BDS-CEC from the predicted CEC calculated using linear regression (y = 0.0044x + 0.604) and HDL-C was compared with serum TP levels. Correlations were evaluated using Pearson’s test. All samples were assayed in triplicate.

To investigate the effects of serum TP levels on BDS-CEC, pooled HDL and lipo-free fraction were prepared by ultracentrifugation and cholesterol and protein levels were determined, respectively. Then, adequate amounts of the pooled HDL and lipo-free fractions were mixed with adequate volumes of PBS to prepare 25 reconstituted serum samples corresponding to 6.0, 6.5, 7.0, 7.5, and 8.0 g/dl of TP, each with five levels of HDL-C (20, 60, 90, 120, and 160 mg/dl). After PEG precipitation, the CEC of BDS (2% in serum) was evaluated. No significant change in CEC caused by changes in TP levels was observed (HDL-C at 20, 60, 90, 120, and 160 mg/dl, with *P*=0.129, 0.362, 0.224, 0.613, and 0.960, respectively, using the Jonckheere–Terpstra test) (Figure 4A). In addition, the correlation was evaluated between CEC deviations, which were defined as the distance of each BDS-CEC from the regression equation obtained by correlation between BDS-CEC and whole-CEC (TC), indicated in Figure 3B, and serum TP levels in 40 patients. CEC deviations and serum TP levels were weakly correlated (Figure 4B, r = 0.319, *P*=0.045). These results indicate that TP level in BDS does not affect its CEC.

### Effects of serum LDL-C and TG levels on BDS-CEC

**Figure 5:**
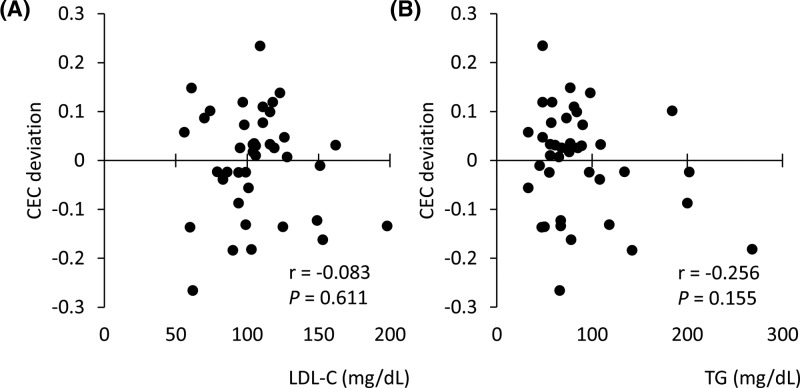
Effects of serum LDL-C and TG levels on BDS-CEC Deviation of BDS-CEC from predicted CEC calculated using linear regression and HDL-C was compared with serum LDL-C (**A**) and TG (**B**) levels. Correlations were evaluated using Pearson’s test. All samples were assayed in triplicate.

To estimate the effects of apoB-containing lipoproteins in the serum, which cannot be excluded as a possible contamination in BDS, the correlation between the CEC deviations described above and LDL-C or TG levels was similarly analyzed. No correlation was observed between CEC deviations and serum LDL-C (Figure 5A, r = 0.083, *P*=0.611) or TG (Figure 5B, r = −0.256, *P*=0.155) levels, suggesting that neither LDL-C nor TG level in BDS affect CEC.

## Discussion

Although CEC has already been reported as a possibly better biomarker than HDL-C to predict the risk of CVD development [5], no analytical method is available for a realistic operation in the clinical laboratory. We developed a novel CEC assay method that is performed without cultured cells or radioisotopes [15]. To complete this method for clinical application, ultracentrifugation should be avoided because of its complexity and time investment. The PEG precipitation method has already been used instead of ultracentrifugation to obtain CA [5,8,13], including the HDL fraction. However, HDL isolated by ultracentrifugation and BDS obtained by the precipitation method are obviously different. Therefore, the present study aimed to compare HDL and BDS as the CA using our novel ILG method. Our results indicate that BDS has a potential as the CA to predict the risk of CVD development in each patient, because CEC obtained using BDS reflects not only the quality but also the quantity of HDL’s function.

In many studies, fixed protein concentration of HDL have been used to evaluate CECs [17–19], although the results of these studies clearly indicate CEC per unit amount of HDL. In our results, no correlations were observed between HDL-C levels and both CECs obtained by the fixed TP (20 μg/ml) and TC (10 μg/ml) concentrations of HDL as a CA. Although HDL-C levels are thought to be insufficient as a risk marker for CVD development [4], nobody denies the inverse correlation between HDL-C levels and a risk of CVD development. CEC as a risk marker for CVD development would therefore at least mildly correlate with HDL-C levels. In addition, CEC per unit amount of HDL is not adequate to estimate the risk of CVD development because HDL levels in patients are not taken into consideration. Some variations observed in the correlation diagram between CECs obtained using the fixed TP and TC concentrations of HDL as the CA are thought to be caused by the different ratio of TP to TC in each HDL, namely, the different profile of HDL subclasses [20,21]. In contrast, whole-CEC (TP) and whole-CEC (TC) showed relatively strong correlation, as if these levels offset the heterogeneity of HDL subclasses.

BDS-CEC was also compared with whole-CECs. A significant correlation was observed between BDS-CEC and whole-CEC (TP) or whole-CEC (TC). These results indicate that BDS can be used as CA reflecting a quantitative CEC of HDL in patients. BDS-CEC could at least strongly assist the role of HDL-C as a risk biomarker of CVD development, because clearly and significantly different BDS-CECs were sometimes observed in patients with similar levels of HDL-C. Conversely, determination of BDS-CEC might be better to evaluate the anti-atherosclerotic status in a patient than measuring HDL-C. However, the effects of serum proteins or contamination of apoB-containing lipoprotein in BDS should be excluded. First, we checked whether TP levels in serum samples affected CEC, because BDS contains not only HDL but also almost all serum proteins. We designed the reconstituted serum to range from 6.0 up to 8.0 g/dl based on the actual protein levels in patients as shown in Table 1. In this range, CEC did not tend to increase or decrease along with TP levels. Even the largest gap of CEC between the reconstituted serum samples with 6.5 and 8.0 g/dl TP in the group of 90 mg/dl HDL-C was 0.1003, which was much lower than the average CEC variation in all HDL-C levels (Figure 3C). For further confirmation, each deviation of BDS-CEC from the regression equation, obtained from the correlation between BDS-CEC and whole-CEC (TC), was compared with the corresponding serum TP levels. TP levels showed a weak correlation with the deviation of BDS-CEC (Figure 4B, r = 0.319, *P*=0.045). However, when only one outlier (5.0 g/dl of TP) was excluded, the significant correlation was lost (r = 0.215, *P*=0.189). These results suggest that the effects of serum proteins on BDS-CEC are negligible.

Next, the effects of serum apoB-containing lipoprotein levels were assessed. Although apoB-containing lipoproteins are thought to be removed by the PEG precipitation method, a small amount of apoB-containing lipoproteins may remain in BDS because this method is not specific for apoB-containing lipoprotein. Remaining apoB-containing lipoprotein could make the CEC higher than the actual function of HDL. Therefore, serum LDL-C and TG levels were also compared with the deviations of BDS-CEC. Neither LDL-C and TG showed a correlation with the deviation, indicating that apoB-containing lipoprotein does not contaminate the BDS, or that the amount of contaminating apoB-containing lipoprotein is too small to affect the CEC.

In conclusion, the present study has demonstrated that CEC obtained using BDS as the CA is a potential new risk marker of CVD development. Moreover, some variations in the correlation between CEC and HDL-C might suggest that HDL-C does not always reflect the function of HDL, which is consistent with a previous report [5]. To confirm an availability of CEC measured by the ILG method using BDS as the biomarker of CVD development, a clinical study with CVD patients is needed. A limitation of the present study is the use of serum from patients whose laboratory data are within the ranges shown in Table 1. Whether serum with highly abnormal clinical data affects CEC assessment or not should also be elucidated. In addition, we also need a reference interval or cut-off value to estimate the CVD risk of a patient.

## Abbreviations

apo: apolipoprotein
apoB: apolipoprotein B
BDS: apoB-depleted serum
BDS-CEC: cholesterol efflux capacity obtained from BDS
CA: cholesterol acceptor
CEC: cholesterol efflux capacity
CVD: cardiovascular disease
HDL: high-density lipoprotein
HDL-C: HDL-cholesterol
ILG: immobilized liposome-bound gel bead
LDL: low-density lipoprotein
LDL-C: LDL-cholesterol
lipo-free: lipoprotein-free
PEG: polyethylene glycol
RT: room temperature
TC: total cholesterol
TG: triglyceride
TP: total protein
